# Immunoinformatics and structural aided approach to develop multi-epitope based subunit vaccine against *Mycobacterium tuberculosis*

**DOI:** 10.1038/s41598-024-66858-5

**Published:** 2024-07-10

**Authors:** Guneswar Sethi, Rinku Polachirakkal Varghese, Avinash Kant Lakra, Subhashree Subhasmita Nayak, Ramadas Krishna, Jeong Ho Hwang

**Affiliations:** 1 Department of Predictive Toxicology, Korea Institute of Toxicology (KIT), Daejeon, Republic of Korea; 2 Animal Model Research Group, Korea Institute of Toxicology, 30 Baehak 1-gil, Jeonguep, Jeollabuk-do 56212 Republic of Korea; 3 Department of Bioinformatics, Pondicherry University, Puducherry, 605014 India; 4 Translational Health Science and Technology Institute, Faridabad, Haryana 121001 India

**Keywords:** *Mycobacterium tuberculosis*, Multi-epitope vaccine, Docking, Molecular dynamics simulation, In silico cloning, Computational biology and bioinformatics, Vaccines, Peptide vaccines

## Abstract

Tuberculosis is a highly contagious disease caused by *Mycobacterium tuberculosis* (*Mtb*), which is one of the prominent reasons for the death of millions worldwide*.* The bacterium has a substantially higher mortality rate than other bacterial diseases, and the rapid rise of drug-resistant strains only makes the situation more concerning. Currently, the only licensed vaccine BCG (Bacillus Calmette–Guérin) is ineffective in preventing adult pulmonary tuberculosis prophylaxis and latent tuberculosis re-activation. Therefore, there is a pressing need to find novel and safe vaccines that provide robust immune defense and have various applications. Vaccines that combine epitopes from multiple candidate proteins have been shown to boost immunity against *Mtb* infection. This study applies an immunoinformatic strategy to generate an adequate multi-epitope immunization against *Mtb* employing five antigenic proteins. Potential B-cell, cytotoxic T lymphocyte, and helper T lymphocyte epitopes were speculated from the intended proteins and coupled with 50 s ribosomal L7/L12 adjuvant, and the vaccine was constructed. The vaccine’s physicochemical profile demonstrates antigenic, soluble, and non-allergic. In the meantime, docking, molecular dynamics simulations, and essential dynamics analysis revealed that the multi-epitope vaccine structure interacted strongly with Toll-like receptors (TLR2 and TLR3). MM-PBSA analysis was performed to ascertain the system’s intermolecular binding free energies accurately. The immune simulation was applied to the vaccine to forecast its immunogenic profile. Finally, in silico cloning was used to validate the vaccine’s efficacy. The immunoinformatics analysis suggests the multi-epitope vaccine could induce specific immune responses, making it a potential candidate against *Mtb*. However, validation through the in-vivo study of the developed vaccine is essential to assess its efficacy and immunogenicity profile, which will assure active protection against *Mtb*.

## Introduction

Tuberculosis (TB) is one of the most ancient diseases that co-evolved with humans for thousands or even millions of years and is considered a global threat to human morbidity^1^. Despite newer modalities for identifying and treating TB, many people are still suffering, making it the second-highest fatal infectious disease^2^. According to recent World Health Organization (WHO) reports, the global incidence of TB escalated to 10.6 million cases in 2022, surpassing earlier projections of 10.3 million in 2021 and 10.0 million in 2020. In 2022, an estimated 1.30 million global TB-related deaths were recorded, approaching 2019 levels following a decline from the peak estimates of 1.4 million in both 2020 and 2021^3^. Notably, the Indian TB Report 2023 delineates a substantial surge, reporting 2.42 million cases in India in 2022^4^. Presently, therapeutic options for TB are limited, predominantly relying on a combination of antimicrobial drugs. Certain first-line drugs, such as ethambutol, isoniazid, pyrazinamide, and rifampicin, have been used to treat the disease caused by *Mtb,* making it more tolerated and multi-drug resistant^5^. The second-line drugs include capreomycin, kanamycin, and amikacin, combined with fluoroquinolones^6^. The BCG is an attenuated form of the vaccine, the only approved vaccine since 1923. However, clinical trials have found conflicting estimates of its effectiveness (ranging from 0 to 80%) in preventing pulmonary TB disease^7^. The variability in the global efficacy of the BCG vaccine is due to genetic heterogeneity in both BCG strains and individuals, as well as interference from persistent parasite infections^6^. Previously, the BCG vaccine offered successful protection against pulmonary TB in childhood but failed against pulmonary TB in adults. The use of BCG as a live-attenuated vaccine in immunocompromised patients rendered high risk as the pathogen can return to its virulent state^8,9^. Several TB vaccines are at the various stages of clinical trials, but safety issues remain a concern, with nearly 50% of these candidate vaccines using a live, attenuated *Mtb* strain^10^. Crucell-Ad35/AERAS-402 and MVA85A, two viral vector-based vaccines, show a subordinate protective efficacy^11^. The potency of recombinant VPM1002 and MTBVAC to revert to a virulent condition is also challenging^12^. Subunit vaccines such as M72 and H4 also contain antigens lacking significant immunogenicity, necessitating multiple vaccinations^13^. Despite the initial success of M72/AS01 E, various participants in phase II clinical studies reported local reactions at the injection site^14^. Developing a peptide vaccine for tuberculosis has gained significant attraction, considering the success of peptide-based vaccines, such as H4/IC31, credited to its stable nature and precise and targeted immune responses. In healthy adults and infants vaccinated with BCG, peptide vaccines have been shown to elicit robust immune responses and to be safe in phase I studies^15^. Based on clinical trial data, subunit vaccines encompass diverse epitopes and have shown a promising effect in the fight against tuberculosis^16,17^. Several investigations have employed immunoinformatic approaches to develop multi-epitope vaccines against tuberculosis^18,19^. In a recent study, Jiang et al. performed an immunoinformatics analysis to develop a multi-epitope vaccine against *Mtb*. The investigation centered on the C624P vaccine, which holds promise as a latent tuberculosis infection (LTBI) preventive measure, showcasing commendable antigenicity, immunogenicity, stability, and the capacity to activate immune responses^20^. In contrast to most of the past research, which uses exosome vesicles or known antigenic proteins to identify epitopes, the current study aims to address the global demand for an effective TB vaccine by developing an in silico multi-epitope vaccine by integrating highly antigenic, non-allergenic and non-toxic epitopes from novel immunogenic proteins.

The literature survey found five antigenic proteins (probable transcriptional regulatory protein, possible exported protein, PPE family protein PPE41, conserved threonine-rich protein, and Phospholipase C A) could serve as vaccine targets. The probable transcriptional regulatory protein is a functional protein of the transcriptional regulation system and regulates the transporter proteins for transporting the fatty acid to the cell wall^21^. It also possesses a strong binding affinity for DNA and is responsible for antibiotic resistance in several disease-causing pathogenic bacteria^21^. The exported proteins of *Mtb* are essential for virulence and bacterial viability^22^. The PE25/PPE41 protein is vital in cellular and humoral responses^23^. It has been observed that the PPE41 protein complexed with PE25 protein generally activates the dendritic cells that induced the proliferation of CD4^+^ and CD8^+^ T cells in the mouse model^24^. The Phospholipase C A protein is primarily involved in virulence and hydrolysis of sphingomyelin and phosphatidylcholine^25,26^. Previous research has also revealed that the enzyme Phospholipase C A may be helpful in the detection of TB and was able to induce a tremendous immune response in mice^27^. The conserved threonine-rich proteins are essential for the survival and spread of the bacterium within the host; they also play a vital role in determining the response of mycobacteria to the environment^28^. These selected proteins of *Mtb* were found to be antigenic and could induce an immune response within the host. These proteins have not been previously explored in the context of vaccine development against *Mtb*, providing a novel avenue for investigation. Additionally, our selection is grounded in a comprehensive antigenicity, non-allergenicity, and non-homology analysis. This deliberate approach aims to identify proteins that exhibit potential immunogenicity, minimize the risk of allergic responses, and offer distinctiveness from known homologous sequences. Therefore, the proteins mentioned earlier were targeted to develop a potential vaccine against *Mtb*, and specific epitopes that can bind with Toll-Like Receptors (TLRs), which can trigger an immunological response inside the host, are selected.

The evolution of sequencing and immunoinformatics has revolutionized drug and vaccine design, replacing traditional, time-consuming approaches with efficient, epitope-based strategies utilizing in silico tools^29^. This advancement enables the efficient identification of therapeutic peptides, propelling the field of immunotherapy forward. The framework of this study is centered on developing a subunit vaccine through the analysis of five antigenic proteins derived from *Mtb*. The resultant chimeric vaccine is composed of 15 cytotoxic T lymphocytes (CTL), 5 interferon-gamma (IFN-γ) inducing helper T lymphocytes (HTL), and 5 B-cell epitopes. The strategic linking of these epitopes, encompassing B-cells, CTLs, and HTLs, is intended to optimize protein folding and enhance flexibility. This multiepitope vaccine, grounded in immunogenic antigens, has been conceived in response to the global tuberculosis pandemic, addressing the imperative for effective TB treatment. The envisaged assessment profile of the vaccine design provides insights into its immunogenicity, laying the foundation for subsequent experimental evaluation.

## Materials and methods

### Sequence retrieval

**Figure 1:**
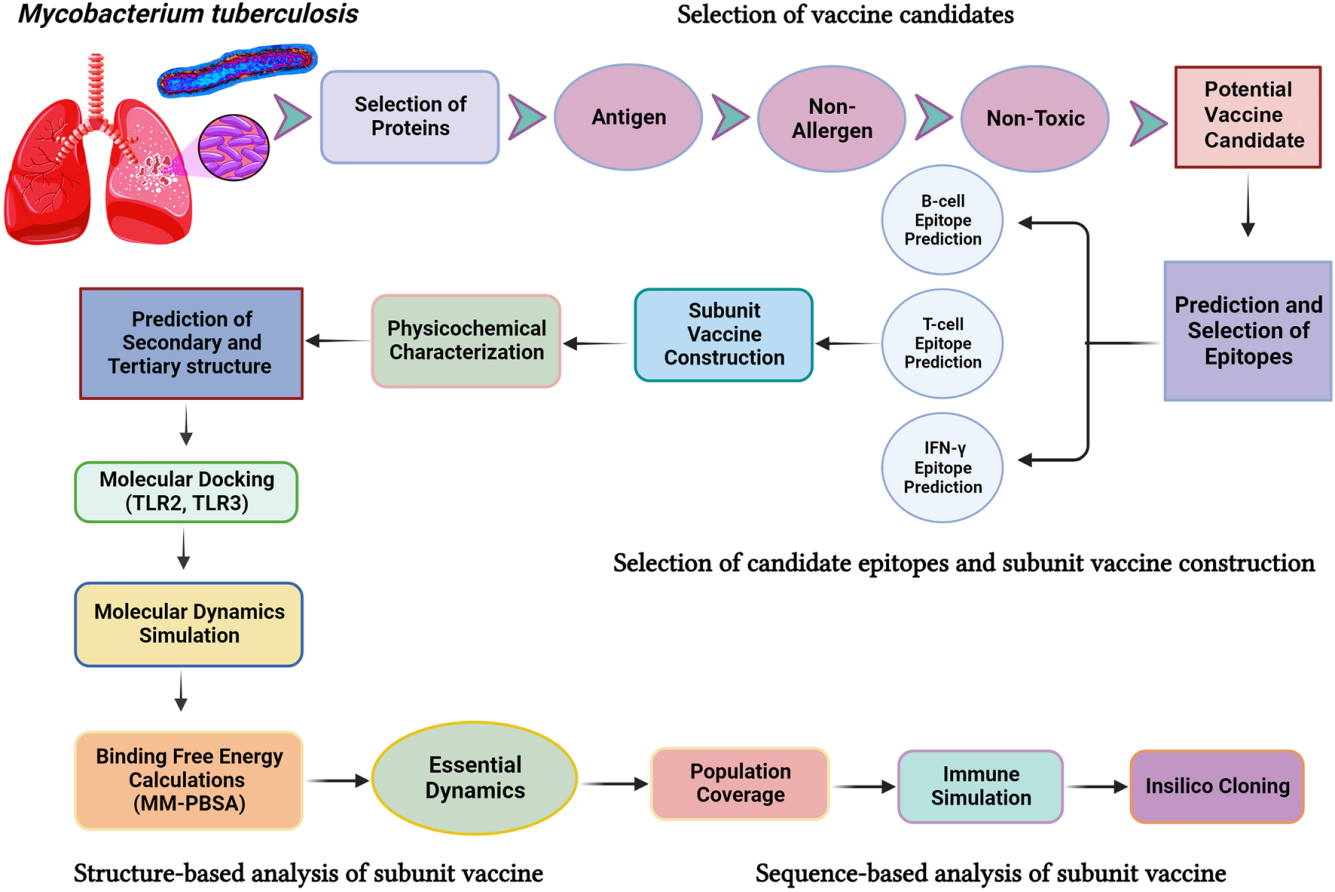
A schematic representation of the workflow used in developing a multi-epitope vaccine against *Mycobacterium tuberculosis*.

The UniProt database^30^ was utilized to retrieve target proteins. Subsequently, the amino acid sequences of specific proteins from *Mycobacterium tuberculosis* (strain ATCC 25618/H37Rv; Proteome ID: UP000001584), namely Probable transcriptional regulatory protein (UniProt ID: P96415), Possible exported protein (UniProt ID: O06567), PPE family protein PPE41 (UniProt ID: Q79FE1), Conserved threonine-rich protein (UniProt ID: I6WZ30), and Phospholipase C A (UniProt ID: P9WIB5), were acquired in FASTA format for further comprehensive analysis. The antigenic propensities were predicted for each selected protein using the Antigenic Peptide Prediction tool^31^. The allergenic property was also discovered using the AllergenFP v.1.0 server, a descriptor-based fingerprint technique^32^. To mitigate the risk of autoimmunity, all chosen proteins underwent scrutiny against the human proteome via the BLASTp tool, utilizing default settings^33^. The methodology for designing the multi-epitope vaccine is illustrated in Fig. 1, and the list of databases, software, and web services used is provided in Supplementary Table 1.

### Prediction of B-cell epitope

Forecasting the B-cell epitope is critical in the vaccine design process; therefore, it has been predicted using the ABCpred server^34^. The server utilizes machine-learning techniques for this prediction. It comprises a dataset of non-repetitive B-cell epitopes retrieved using the Bcipep database and random non-epitopes obtained from the Swiss-Prot database with an accuracy of 65.93%^35^. The window length of 16 residues and a scoring threshold value of 0.8 were set for this prediction. The best-scoring epitopes, with a score > 0.8, were selected and used in further study.

### Prediction of Helper T-lymphocyte epitope

The following step assesses the IEDB MHC II server to evaluate the HTL epitopes of the chosen proteins^36,37^. In this study, the epitopes were projected using default parameters, considering a reference set of alleles, as they represent 99% of the total allele distribution. Subsequently, the HTL epitopes with the lowest percentile rank were selected for further investigation.

### Prediction of potential Cytotoxic T-lymphocyte epitope

The NetCTL 1.2 server was used to assist in predicting CTL epitopes using antigenic proteins^38^. Herein, the integrated methods of proteasomal cleavage, TAP transport efficiency, and MHC class I binding are used to identify epitopes. For the epitope forecasting, 3 HLA class I supertypes were assessed: A2, A3, and B7; they present an overall population coverage of 86%^39,40^. We selected 9-mer peptides according to their highest score against each protein sequence in this analysis.

### Identification of IFN-γ inducing epitope

IFN-γ is a cytokine crucial in creating and regulating different immune responses. Therefore, the prediction was made using the IFNepitope server for the selected HTL epitopes^41^. The forecast was created using the motif and SVM hybrid method, and the model selected for the prediction was IFN-γ versus other cytokine models.

### Prediction of toxicity

The selection of a non-toxic epitope is necessary for the development of a subunit vaccine. Therefore, the ToxinPred server was used to assess the toxicity of all predicted epitopes^42^. The epitopes that passed the non-toxic filters were subsequently employed to develop the vaccine.

### Construction of final vaccine and physicochemical characterization

All epitopes predicted in the previous phases were combined with an adjuvant and linker to generate a subunit vaccine. This method connected CTL, HTL, and BCL epitopes via AAY, GPGPG, and KK linkers. Herein, linkers were applied to separate the epitopes effectively, and the construct’s immunogenicity was enhanced by using an adjuvant 50 s ribosomal L7/L12 (P9WHE3) linked by an EAAAK linker^43^.

The physicochemical properties of the vaccine were evaluated using the Expasy-ProtParam web server^44^. An aliphatic index, molecular weight, half-life, GRAVY, and theoretical pI were used to predict these properties. The antigenicity of the protein sequences was predicted by VaxiJen v2.0^45^. The threshold value for prediction accuracy was set at 0.4, and the target organism chosen was a bacterium. This server’s prediction accuracy ranges from 70 to 89%. The designed construct was further tested for antigenicity using a sequence-based server called ANTIGENpro^46^. A SOLPro server employing the SVM architecture predicted how soluble a protein would be based on overexpression in *E. coli*^47^. AllerTOP v2.0 was used to indicate the allergenicity of the developed vaccine, with an accuracy of 88.7%^48^.

### Structure determination and validation

The three-dimensional structures of the selected proteins were predicted using the AlphaFold program (https://alphafold.ebi.ac.uk/) to identify optimal epitope positions within the structure^49^. The PSIPRED v4.0 and GOR IV web servers, which utilize neural networks and information theory, were employed to predict the alpha helices, beta sheets, and coils of the anticipated vaccine^50,51^. The RaptorX web tool was employed to understand the vaccine’s tertiary structure^52^. It predicts protein structure using the template approach and ranks the models based on their *P* values. The vaccine structure was refined by a web server called GalaxyRefine^53^. The server is built on an ingenious procedure that performs reassembling and structural refinement using molecular dynamics modulation. The structural validation of the vaccine was conducted through the analysis of the Ramachandran plot via PROCHECK^54^ and ProSA-web^55^. Finally, the 3D structure of the vaccine was further visualized using Chimera 1.17.1^56^.

### Molecular docking

Molecular docking is a computational method widely employed to predict the binding interactions and affinities between molecules with known structures^57^. The docking was performed by a ClusPro 2.0 server^58^, which improved the contact between the antigen and the receptors TLR2 (PDB ID: 4G8A) and TLR3 (PDB ID 5GMF). The complexes were graphically represented for binding with the aid of the UCSF Chimera 1.17.1 software^59^. We choose the one with the least global binding energy to assess intermolecular interactions. Finally, the PDBsum tool was employed to predict the interactions between each complex^60^.

### Molecular dynamic simulation (MDS)

For stability analysis of the proposed vaccine construct with the presence of the TLRs, the MDS was carried out with the help of GROMOS 9643a1 force fields of GROMACS 2019^61^. Three systems were prepared for MDS: a free vaccine and vaccines complexed with TLR2 and TLR3. Next, we generated the topology, which is the first step as it provides all the details about the non-bonded and bonded parameters required to determine the structure of the docked complex within a simulation. Each system was solvated in a cubic box with water molecules (SPC) and neutralized by introducing suitable ions before simulation. To mitigate the initial steric collisions, the complexes were energy minimized by 100 ps using the steepest descending method. Further, the entire system was equilibrated in NVT and NPT ensembles with 100 ps at 300 K and one pressure bar. The Particle-Mesh Ewald electrostatics (PME) method was used for handling the van der Waals and electrostatic interactions, with a cut-off of 1 nm^62^. Further, the bond length and water geometry constraints were enforced through the LINCS and SETTLE algorithms^63,64^. The weakened Berendsen thermostat was used for temperature regulation, while the Parrinello-Rahman approach was applied for pressure regulation^65^. Subsequently, the MDS was conducted for 100 ns to determine the complex’s RMSD, RMSF, Rg, and H-bond^66^. Following MDS, the binding free energy of the complexes was determined using the GROMACS platform’s g_mmpbsa v5.12 program.

### Essential dynamics (EDs)

To gain insight into the dynamics and functional motions of both complexes, the EDs were conducted on the Cα atoms^67^. The first step in this method was to create a covariance matrix for Cα variations. The covariance matrix was then diagonalized to provide information regarding the precise movement of the molecule with the help of eigenvectors (EVs) and eigenvalues. EVs indicate the direction of the molecule’s movement, while eigenvalues represent the magnitude of the collective movement of the molecules^68^. Further, the covariance matrix and EVs of the first two principal components (PCs), PC1 and PC2, were constructed using the GROMACS commands gmx covar and gmx anaeig. Furthermore, RMSD-based clustering analysis with a cut-off value of 0.25 nm was used to investigate the structural variability of complex systems. The analysis used the GROMACS' gmx cluster framework^68^.

### Vaccine population coverage

Varying populations around the world have different HLA genotype frequencies. Therefore, the type of HLA polymorphism affects how a specific peptide from the multi-epitope vaccine binds to HLA alleles^69^. Thus, confirming the degree to which the vaccine created in this study has shielded the entire world’s population is necessary. The prioritized vaccine sequences were studied using the IEDB population coverage tool^69^ using default parameters to ascertain the population response.

### Codon optimization

The JCat server was employed for reverse translation and codon optimization of the vaccine sequence to achieve maximum expression in *Escherichia coli*^70,71^. The server ensured the best expression, and the vaccine’s GC content percentage and CAI scores were recorded. The vaccine was subsequently cloned into the pET28a (+) vector, and *XhoI* and *BamHI* restriction sites were added to the N and C-terminals of the sequence, respectively. Finally, the SnapGene tool was used to clone the vaccine.

### Host-immune system simulation

The vaccine construct was further analyzed using the C-ImmSim server to simulate the natural immune response within the human body^72^. It visually represents a mammalian system’s humoral and cellular reactions to a vaccine constructed using PSSM and machine learning techniques. The vaccine was administered in doses with intervals of 4 weeks. Simulation parameters were set at intervals of 1, 84, and 168. We kept the simulation volume at 50, and the simulation steps were 1000 (LPS-free vaccination injection, random seed = 12.345) for this vaccination.

### Ethical approval

This article contains no studies with human participants and animals performed by authors.

## Results

### Protein sequence retrieval

In this study, five immunogenic proteins of *Mtb* (H37Rv strain) were selected from the UniProt database, and immunogenic prediction and physicochemical features analysis were performed for vaccine construction. Following the investigation, we selected Probable transcriptional regulatory protein (antigenic score: 1.0405), Possible exported protein (antigenic score: 1.0315), PPE family protein PPE41 (antigenic score: 1.02841), Conserved threonine-rich protein (antigenic score: 1.009), and Phospholipase C A (antigenic score: 1.316), for epitope antigenicity prediction (Supplementary Fig. 1). In a subsequent assessment through the AllergenFP v.1.0 server, all identified proteins demonstrated a non-allergenic profile (Table 1). All selected proteins were non-homologous after conducting a non-homology search against the host proteome. Subsequently, based on this allergenicity evaluation, these proteins underwent additional scrutiny for the strategic design of a multi-epitope vaccine.

**Table 1:** Selected *Mycobacterium tuberculosis* proteins used to develop subunit vaccine design.

Protein name	UniProt ID	Antigenic propensity	Protein Length (A.A)	AllergenFP prediction	Non-homologous	Function
Probable transcriptional regulatory protein	P96415	1.0405	229	Non-allergen	✓	Involved in transcriptional mechanism^21^
Possible exported protein	O06567	1.0315	232	Non-allergen	✓	Crucial for both bacterial viability and virulence^22^
PPE family protein PPE41	Q79FE1	1.0284	194	Non-allergen	✓	As a dimer, the protein induces both strong humoral and cellular immune response^23^
Conserved threonine rich -protein	I6WZ30	1.009	165	Non-allergen	✓	Critical for the bacterium’s survival and proliferation within the host^28^
Phospholipase C A	P9WIB5	1.0316	520	Non-allergen	✓	Participates in virulence and breakdown of sphingomyelin and phosphatidylcholine^25,26^

### Epitopes prediction

To develop a vaccine that precisely represents the immune response generated by infection while creating long-lasting adaptive immunity, epitopes from the five target proteins were identified by the ABCpred server. B-cell epitopes play a crucial role in adaptive immunity because B-cell receptors recognize them and generate immunoglobin in response^73^. Hence, five epitopes with the highest score and 16 mer window lengths were selected for each protein (Table 2).

**Table 2:** B-cell epitopes prediction for the input *Mycobacterium tuberculosis* protein sequences.

Si. No	Protein name	Sequence	Start position	Score
1	Probable transcriptional regulatory protein	PLVRDAHARGDLRADS	142	0.90
2	Possible exported protein	CPTQRPPVTPRHNLCN	171	0.97
3	PPE family protein PPE41	SANIYAGPGPDSMLAA	12	0.94
4	Conserved threonine-rich protein	SIQIGDMLTYGSIGTT	73	0.92
5	Phospholipase C A	SWRIMPENLEDAGVSW	226	0.96

CTLs are vital for immune system activation. Their involvement in vaccine design is promising as they enable MHC class I molecules to present short epitopes on T-cell surfaces after protein processing^74^. To predict CTL epitopes for each protein sequence, the NetCTL 1.2 server was used, which assigns a score to each predicted epitope. The high score of the epitopes shows that they have an excellent binding affinity and low sensitivity. In total, 15 CTL peptides were picked from these five proteins and further analyzed (Table 3).

**Table 3:** CTL epitopes prediction for the input *Mycobacterium tuberculosis* protein sequences.

Si. No	Protein name	Epitopes					
		A2	Score	A3	Score	B7	Score
1	Probable transcriptional regulatory protein	ALMSLLLLI	1.3576	SLFQYFADK	1.52	HPRERALHA	1.4706
2	Possible exported protein	ALATLLTLI	1.216	GFSTSLLAK	0.871	MPGALSAAL	1.5552
3	PPE family protein PPE41	WLTDLCVQL	1.2129	ALSKLTPWK	1.5204	TPWKAPPPI	1.3958
4	Conserved threonine-rich protein	ALAAAALPL	1.2657	GLDNTVTYK	1.315	RATHSRLAT	1.2671
5	Phospholipase C A	YLLADTFTI	1.5426	MSRREFLTK	1.3358	MPTQETTPV	1.7158

The function of helper T-cells is essential for the successful induction of acquired immunity or adaptive immunity. Additionally, the epitopes of HTL assist the B-cell in generating and releasing antibodies. Furthermore, it facilitates the CTL in eliminating infected cells^75^. Considering the crucial function, we envisaged HTL epitopes supporting the B-cell and T-cell epitopes in stimulating a robust immune response. The top five HTL epitopes (i.e., DLYAFIADIASQRVR, GALSAALAAAAPVWP, DEDGEVMRDYRLRVS, ATFALALAAAALPLA, and AVSMVTALRILLSNP) were chosen according to their lowest percentile ranking, subsequent analysis discovered all epitopes to be non-allergenic, non-toxic, antigenic, and IFN-*γ* positive (Table 4).

**Table. 4.** Selected HTL epitopes for the input *Mycobacterium tuberculosis* protein sequences and their IFN-γ inducing properties.

Si. No	Allele	Epitope	Method	Percentile rank	IC_50_	IFN-γ inducer	
						Result	Score
1	HLA-DRB1*03:01	DLYAFIADIASQRVR	Consensus (smm/nn/sturniolo)	0.06	26	Positive	0.41
2	HLA-DQA1*05:01/DQB1*03:01	GALSAALAAAAPVWP	Consensus (smm/nn/sturniolo)	0.03	9.5	Positive	0.409
3	HLA-DRB1*03:01	DEDGEVMRDYRLRVS	Consensus (comb.lib./smm/nn)	0.91	26	Positive	31
4	HLA-DRB1*09:01	ATFALALAAAALPLA	Consensus (smm/nn/sturniolo)	0.18	32	Positive	0.44
5	HLA-DQA1*01:02/DQB1*06:02	AVSMVTALRILLSNP	Consensus (smm/nn/sturniolo)	0.01	12	Positive	4

### Final vaccine construction, structure, and physiochemical properties estimation

**Figure 2:**
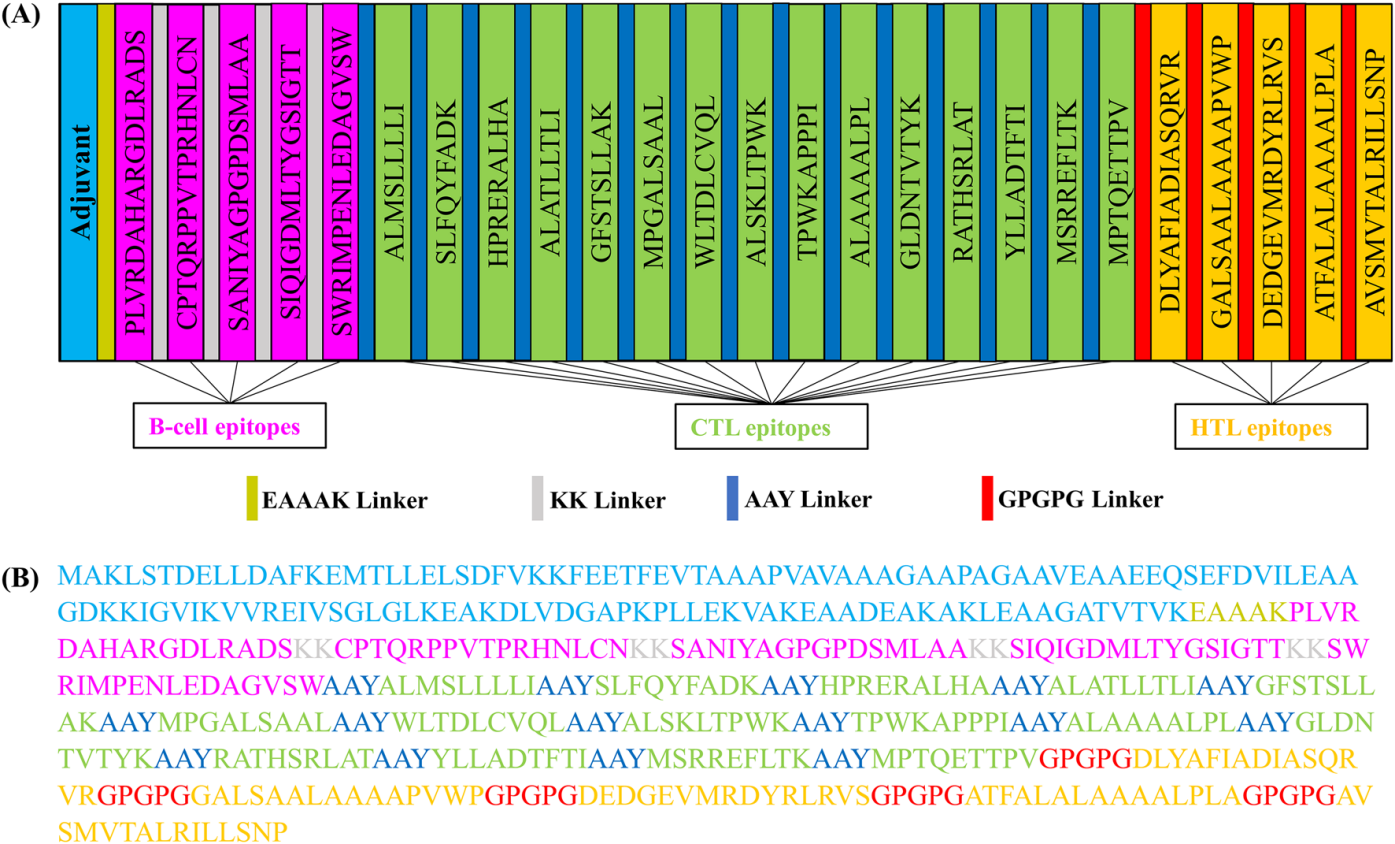
(**A**) Schematic representation of the final vaccine construct components. (**B**) The primary sequence of the vaccine construct is where B-cell, CTL, and HTL epitopes are shown in pink, light green, and gold colors, respectively. A vaccine adjuvant (light blue) is added to the N-terminus of the sequence by an EAAAK linker (dark yellow). The B-cell, CTL, and HTL epitopes are joined by KK (grey), AAY (blue), and GPGPG (red) linkers, respectively.

The vaccine design was created by connecting 15 CTL, 5 HTL, and 5 B-cell epitopes with their appropriate linkers and adjuvants. 50 s ribosomal protein L7/L12, an adjuvant, was integrated into the vaccine design at terminus N through a linker called EAAAK. Finally, we designed a 503 amino acid linear vaccine construct (Fig. 2).

**Figure 3:**
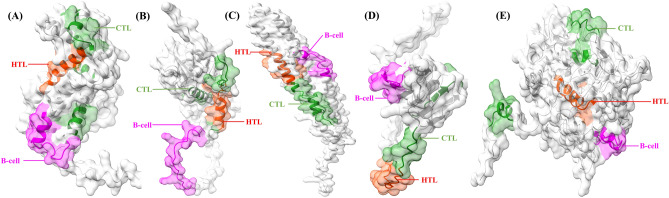
The three-dimensional structure predicted by the AlphaFold program for the selected antigenic proteins (**A**) Probable transcriptional regulatory protein, (**B**) Possible exported protein, (**C**) PPE family protein PPE41, (**D**) Conserved threonine-rich protein, and (**E**) Phospholipase C A with B-cell epitopes marked in magenta, whereas CTL and HTL epitopes were marked in forest green and orange-red respectively.

The epitope sequences included in the vaccine were mapped onto the 3-D structures of their respective selected proteins, demonstrating that the epitopes retained their original positions (Fig. 3). Additionally, detailed structural information of the predicted proteins was highlighted in Supplementary Fig. 2.

**Figure 4:**
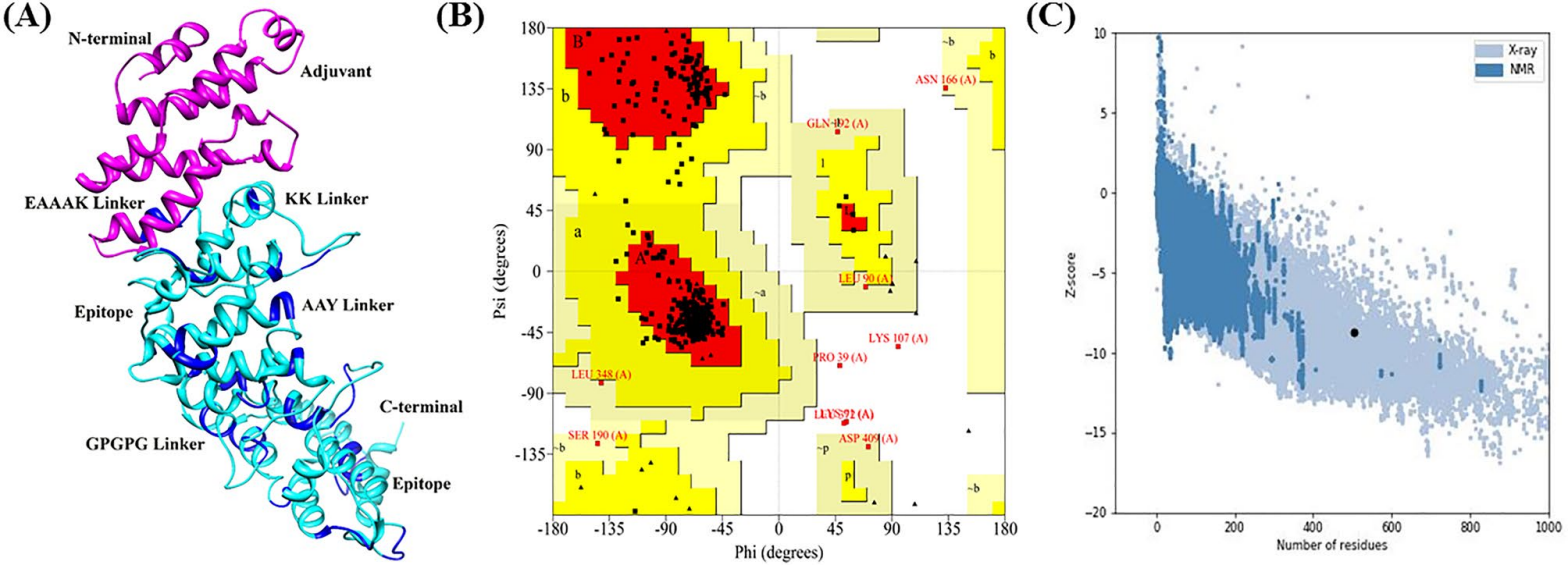
The diagram depicts the final three-dimensional structure of the subunit vaccine, refinement, and model validation. (**A**) 3D vaccine structure predicted through Chimera 1.17.1 (**B**) The Ramachandran plot of the improved vaccine structure (**C**) The overall model quality of the refined structure analyzed through the ProSa server using Z-score.

The GOR IV analysis indicated that the vaccine structure consists of 59.44% α-helix, 8.95% β-strand, and 31.61% coil. Supplementary Fig. 3A,B show the graphical representation of the secondary structure as the GOR IV and PSIPRED servers predicted, respectively. The 3D structure of the vaccine construct was generated using the RaptorX server. Five structures were produced by the server using target sequence ab initio modeling. The one with the least P-value was selected as the best mode. In addition, the selection of the chosen tertiary structure was refined with the help of the GalaxyRefine web server. The Ramachandran plot was produced to assess the general quality of the modeled structure. In the refined model, 93.74% of residues are in the preferred region, compared to 89.1% in the unrefined structure. In conclusion, the Z-score of the improved structure was found to be − 8.6, as determined by the ProSA-web server (Fig. 4).

In addition, the construct’s molecular weight, which was found to be 52.72 kDa, was also verified before further investigation, and the isoelectric point score was 8.15. The intended vaccine sequence was discovered to be an ideal antigen with a score of 0.5687 and 0.5808 predicted using the Vaxigen V2.0 and the ANTIGENpro servers, respectively. The construct was soluble following overexpression, with a score of 0.9434, and was found to be non-allergenic through AllerTOP v. 2.0. Additionally, it was discovered using the ProtParam tool that the construct also matched all other characteristics, like the aliphatic index of 94.04, instability index of 29.63, and grand average hydropathicity of 0.157 (Supplementary Table 2). The vaccine construct instability index shows that the protein is stable upon expression. Furthermore, the aliphatic index represents the thermo-stable nature of the construct, while the GRAVY score indicates its hydrophilic nature. Furthermore, the thermo-stable nature of the construct was represented by its aliphatic index, and the hydrophilic nature was represented by its GRAVY score.

### Molecular docking analysis

**Figure 5:**
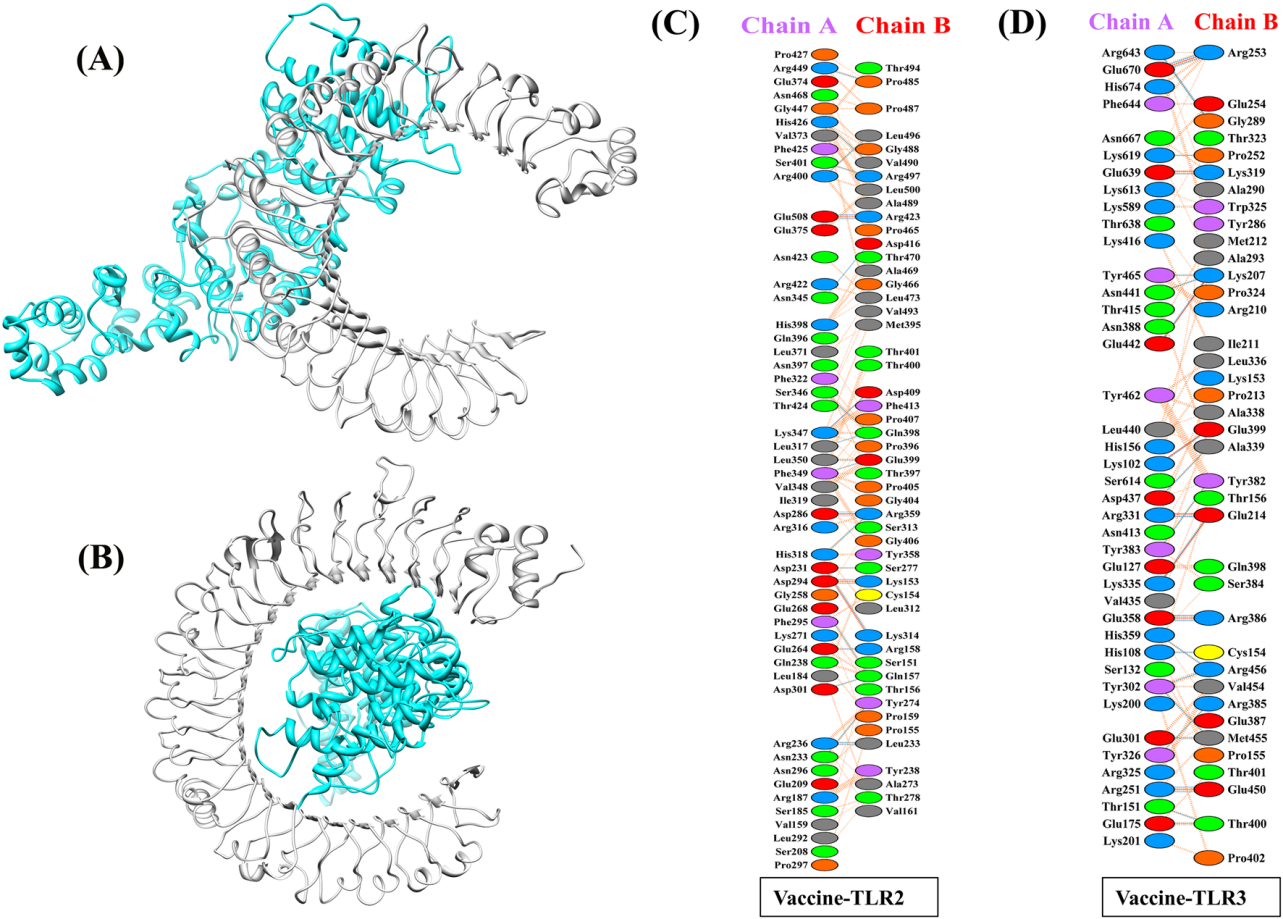
Molecular docking of the vaccine construct (cyan) with immune receptors (light grey). (**A**) The cartoon depiction of the vaccine-TLR2 complex and (**B**) the vaccine-TLR3 complex are illustrated using Chimera software. (**C**) Bond interactions between TLR2 (chain-A) receptor residues and vaccine (chain-B) residues. (**D**) Bond interactions between TLR3 (chain-A) receptor residues and vaccine (chain-B) residues.

Understanding the molecular specifics of antigen detection is a vital and practical step in successfully developing peptide-based vaccines^76^. The vaccine must interact with target immune cell receptors known as TLRs to elicit a sustained immunological response. TLRs are important for finding specific molecules in the body and play a significant role in the natural defense system^77^. We used molecular docking to see how the vaccine would interact with these immune molecules. The docking results revealed that the suggested vaccine binds to the TLR2 receptor more robustly (− 1262.0 kcal/mol) than the TLR3 receptor (− 1162.9 kcal/mol). We used Chimera software to visualize the bonded complex of the TLR2 (Fig. 5A) and TLR3 (Fig. 5B) with the vaccine, and then we used PDBSum to see how the residues between the vaccine-TLR2 (Fig. 5C) and the vaccine-TLR3 interacted (Fig. 5D). According to interaction analysis, the proposed vaccine has five salt bridges, twenty-five hydrogen bonds, and 269 non-bonded contacts with TLR2. On the other hand, it shows 26 hydrogen bonds, 8 salt bridges, and 209 non-boned interactions with TLR3. The docking results demonstrated that the vaccine strongly interacts with the immune receptor for significant immunological reactions.

### Molecular dynamics simulation, essential dynamics, and MM-PBSA analysis

**Figure 6:**
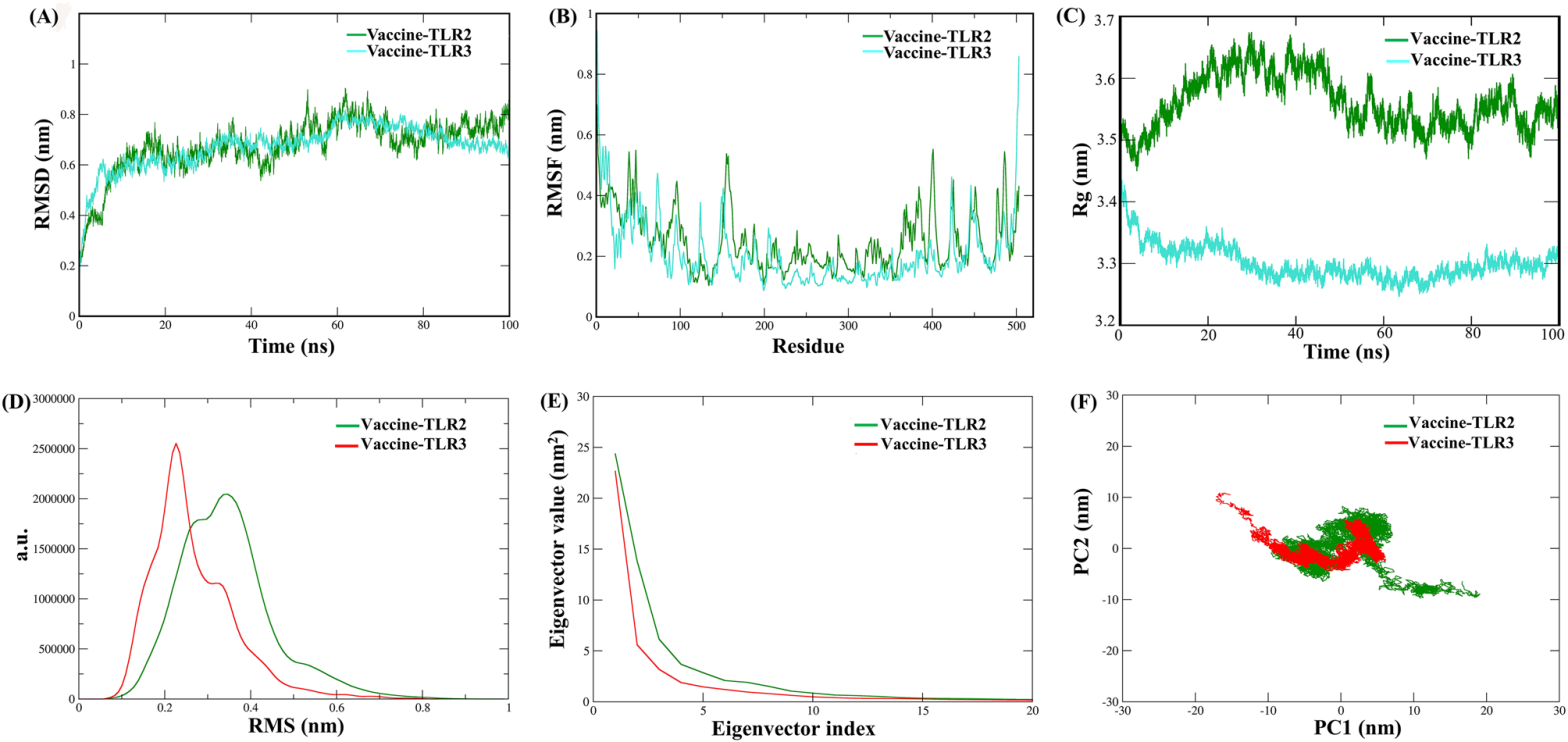
Molecular dynamic simulation and EDs analysis of the vaccine receptor complexes. (**A**) Root mean square deviation plot for the vaccine-TLR2 and vaccine-TLR3 complexes, (**B**) Root mean square fluctuation of the vaccine-TLR2 and vaccine-TLR3 complexes, (**C**) Radius of gyration analysis between the vaccine-TLR2 and vaccine-TLR3 complexes, (**D**) Cluster analysis with Cα-RMSD with a cutoff of 0.25 nm for the docked complexes obtained from a 100 ns simulation, (**E**) Eigenvalues for the complex as a function of the first 20 eigenvectors, (**F**) 100 ns simulation trajectories projected onto the first two principal components (PCs), with the x-axis and y-axis representing PC1 and PC2, respectively.

To comprehend the dynamic motion and stability of the developed vaccine complex, an MDS was conducted for 100 ns. The stability of the docked complexes was estimated in terms of RMSD, RMSF, and Rg over the simulation period. The RMSD graph for the TLR2-vaccine complex exhibits modest deviations between 0.23 and 0.85 nm but attains stability after 70 ns. The TLR3-vaccine complex displayed good stability at 0.6 up to 0.75 nm, indicating a minor variation after 50 ns, as shown in Fig. 6A. RMSF analysis was employed to evaluate the flexibility and stability of the complexes. The TLR2-vaccine complex showed modest fluctuations, with maximum and minimum RMSF values of 0.95 and 0.85 nm, respectively (Fig. 6B). Similarly, the TLR3-vaccine complex exhibited maximum and minimum RMSF values of 0.7 and 0.42 nm (Fig. 6B). Rg analysis also measured structural equilibrium and protein compactness throughout the simulation period. As depicted in Fig. 6C, the average Rg value observed during the simulation run for the vaccine-TLR2 complex was 3.5 nm^2^, while for the vaccine-TLR3 complex, it was 3.3 nm^2^. Furthermore, hydrogen bond analysis revealed that vaccine-TLR2 and vaccine-TLR3 produced an average of 20 and 22 H-bonds, respectively (Supplementary Fig. 4).

The ED analysis was conducted to comprehend the docked complexes overall motion and conformational alteration. The cluster analysis revealed that for the vaccine-TLR2 complex, the RMSD was located between wavelengths between 0.0592077 and 0.989433 nm, with an average value of 0.170811 nm. The cluster accounted for approximately 55% of the total structure (Fig. 6D). For the vaccine-TLR3 complex, the cluster comprised about 48% of the overall structure, and the RMSD ranged from 0.0576789 to 0.838208 nm (with an average value of 0.167755 nm). Further, the diagonalization of a covariance matrix results in a sequence of eigenvalues plotted in descending order from the corresponding eigenvector indices. A plot of the first 20 eigenvalues versus their respective index has been generated (Fig. 6E).

The graph shows that the first few eigenvalues of each eigenvector rapidly decrease in amplitude, leading to a range of restricted values that indicate more considerable regional variations. We found that the top 20 eigenvectors accounted for more than 95% of the movement, and we made an elbow plot by comparing the eigenvalues to the indices. By projecting collected trajectories onto the first two PCs, the scatter plot was constructed, which displays the general motion of the systems as varied in nature (Fig. 6F).

The MM-PBSA analysis was also utilized to confirm the stability of the chosen complexes throughout the MD run. The MM-GBSA values for both the vaccine-TLR2 and vaccine-TLR3 complexes are provided in Table 5.

**Table 5:** The energy components of the vaccine-receptors (TLR2 and TLR3) complex, including electrostatic, van der Waal, polar solvation, SASA, and binding energy, were estimated using the MMPBSA analysis.

Energy type (kJ/mol)	Vaccine-TLR2	Vaccine-TLR3
Electrostatic energy	− 2388.270 ± 4.447	− 2893.054 ± 7.172
van der Waal energy	− 631.509 ± 1.182	− 1031.951 ± 1.425
Polar solvation energy	1620.573 ± 6.170	2357.881 ± 7.367
SASA energy	− 81.834 ± 0.173	− 146.826 ± 0.196
Binding energy	− 1480.962 ± 4.633	− 1714.227 ± 3.227

### Immune simulation

**Figure 7:**
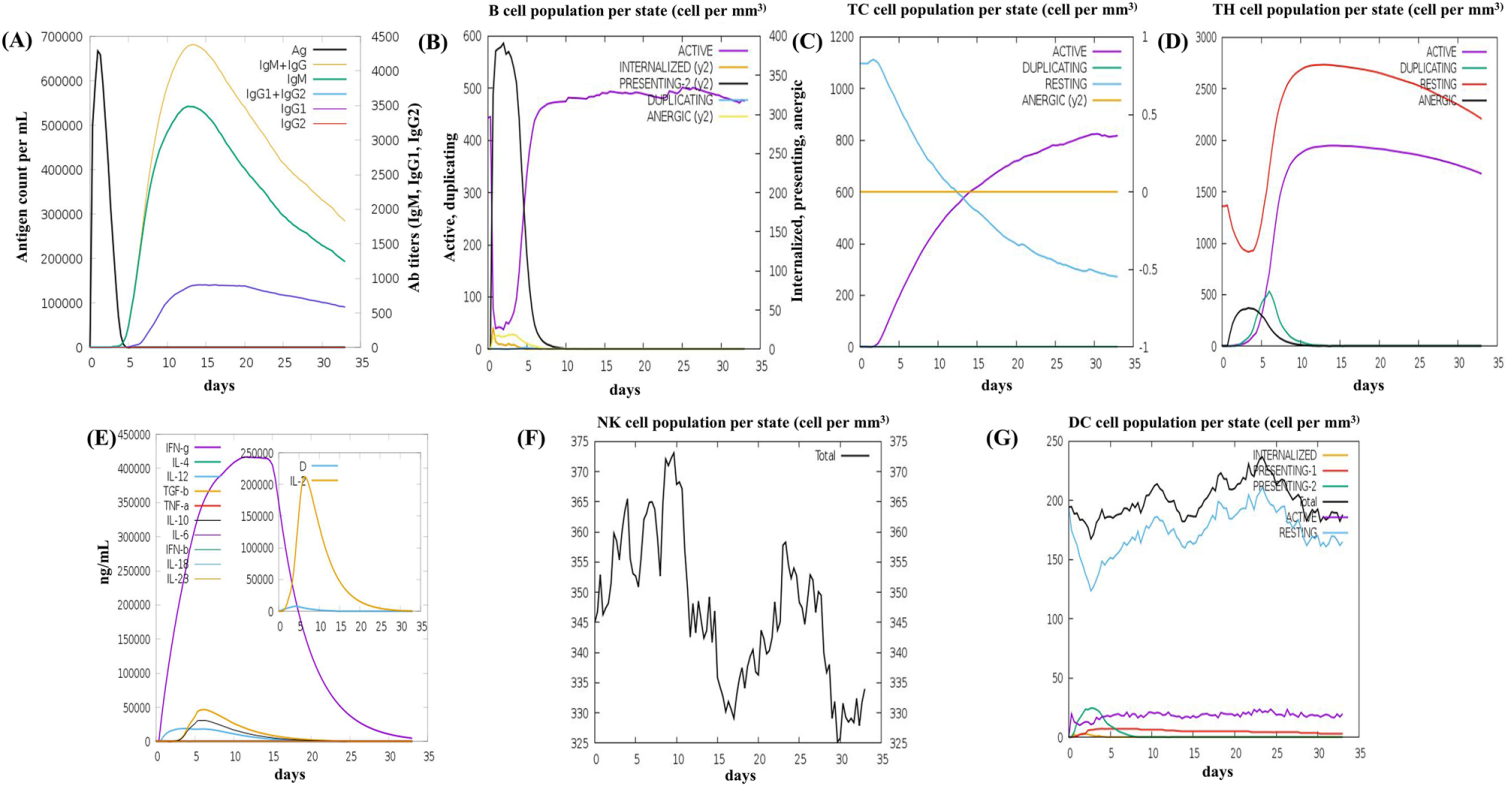
In silico immune simulation results of the vaccine construct using C-ImmSim are presented as follows: (**A**) Immunoglobulin response to antigen injection, with various immunoglobulin subclasses represented by colored peaks; (**B**) Active B-cell population observed following vaccine administration; (**C**) Production of cytotoxic-T cells in response to the vaccine; (**D**) Emergence of Helper-T cells; (**E**) Graph showing the cytokine levels induced by the vaccine, with the inset graph illustrating the Simpson Index, D for IL-2, which measures diversity; (**F**) NK-cell population; and (**G**) Dendritic cell population per state.

The vaccine antigen was administered to the host body, and our vaccine design elicited robust primary and secondary immune responses, as depicted in the graph (Fig. 7). The primary immune response, characterized by an elevation in IgM antibodies, manifests after a lag time of five to seven days following antigen exposure. Subsequently, the secondary immune response is marked by elevated expression of IgM, IgG1, and IgM-IgG antibodies, accompanied by increased B-cell proliferation (Fig. 7A,B). Simultaneously, the population of cytotoxic (Tc) and helper (Th) cells demonstrates a clear increase post-immunization, as depicted in Fig. 7C,D. Furthermore, the enhanced production of IFN-γ and IL-2 observed after immunization underscores the activation of essential immune effectors (Fig. 7E). The assessment of clonal specificity using the Simpson index, D, suggests a potentially diverse immunological response. Prior research highlights innate immune cells, such as NK cells and DCs, in combatting *Mtb*^78,79^. Subsequent analysis revealed a potent activation of these cells, particularly a rapid and sustained increase in NK cell population, which peaked on day 5 (374 cells/mm^3^) post-primary immunization, followed by subsequent fluctuations around (345–325 cells/mm^3^) (Fig. 7F). Resting DCs played a key role in determining the total counts of DCs (Fig. 7G). Post-vaccination, resting DCs increased from 150 to approximately 200 cells/mm^3^ while inducing limited proliferation of presenting-1 and presenting-2 DCs. These results collectively signify a substantial and comprehensive immune response against *Mtb*, validating the efficacy of our vaccine design.

### Population coverage analysis

Population coverage was performed using the vaccine and their respective HLA alleles to predict the response of different world populations. The study reveals that the vaccine shows a world population coverage of 98.76%, where Europe has the highest population coverage (99.7%), followed by North America (99.26%) and the West Indies (98.95%) (Supplementary Table S3). The region with the least population coverage is Central America (12.34%) (Supplementary Fig. 5). Consequently, the vaccination was anticipated to be efficacious across various genetic origins.

### Optimization of codons and in silico cloning

**Figure 8:**
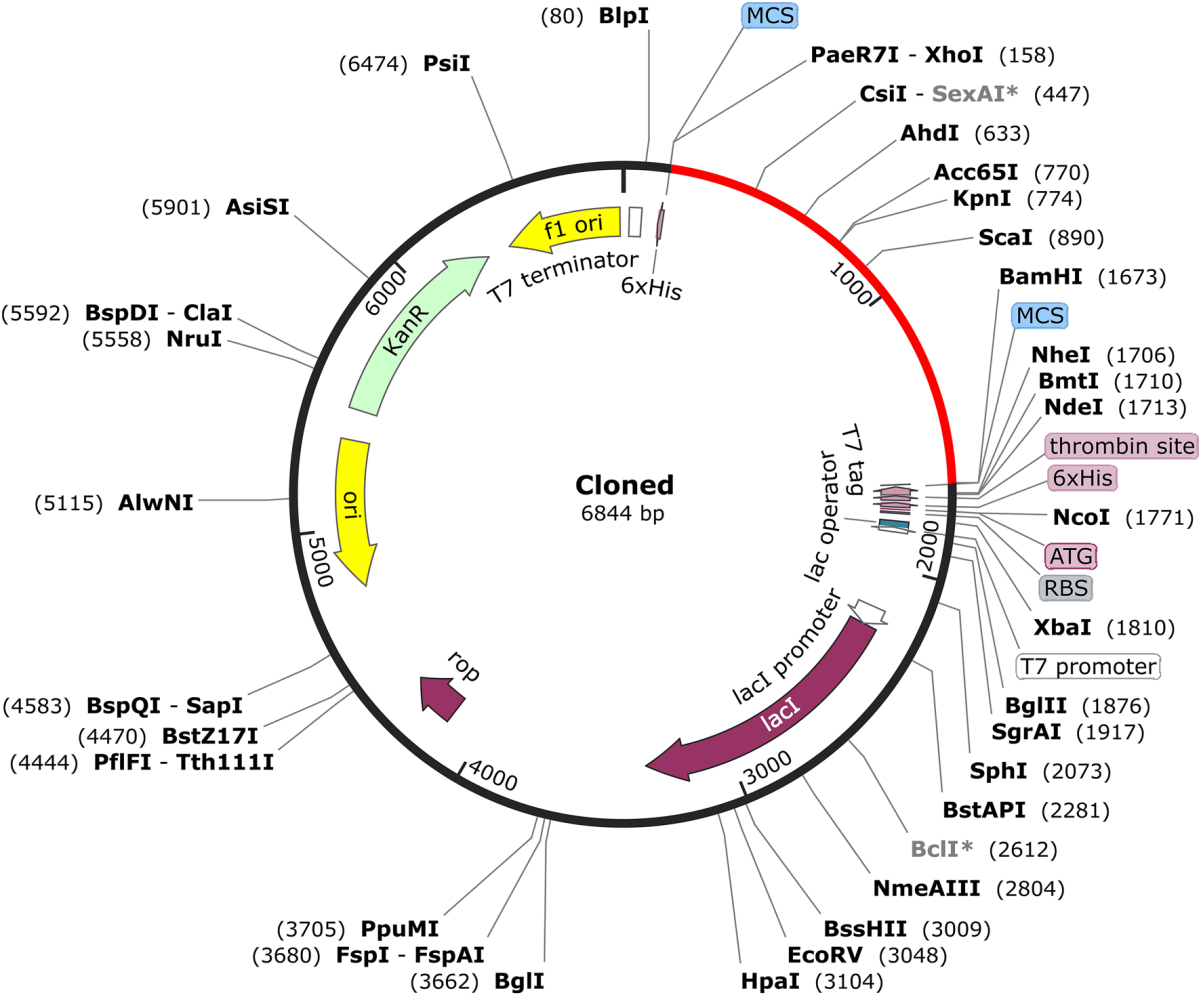
In silico cloning was employed to insert the vaccine design into a pET28a (+) expression vector using *XhoI* and *BamHI* restriction sites. The vaccine-inserted area is highlighted in red, while the vector (pET28a) is depicted in black.

Achieving maximum vaccine expression is particularly crucial in experimental research, where the codon optimization of the amino acid sequence depends on the host expression system^80^. The vaccine was codon-optimized for expression on *E. coli*, yielding a sequence of 503 amino acids (1509 bp). The CAI and GC content of the vaccine are within the optimal range of 0.98 and 55.59%, respectively. Additionally, the restriction enzymes *XhoI* and *BamHI* were chosen and appended to the start and end of the vaccine sequence, respectively. Using the SnapGene tool, the cloning process was computerized by inserting cDNA into a pET28a (+) vector (Fig. 8).

## Discussion

*Mtb* is the foremost cause of death among the global population and is well-known for its resistance to antibiotic treatment^81^. Currently, the only form of tuberculosis vaccine available is a BCG vaccine, which has been demonstrated to be marginally effective against pediatric additional pulmonary tuberculosis but is not effective against pulmonary tuberculosis, the most common form of adult Tuberculosis^82^. The various BCG culture procedures used by multiple laboratories over the past few decades can be attributed to variances in the effectiveness of BCGs, eventually leading to genetic differences across BCG strains^83^. The inability of BCG to fully protect against Mycobacteria can be explained by prior exposure to standard environmental Mycobacterium and the induction of short-lived T effector memory cells. Compared to *Mtb*, BCG also lacks several vital antigens in the Region of Deletion (RD) region^84^. Over the years, the scientific community has diligently identified numerous promising vaccine candidates poised to replace the existing BCG vaccine^85–87^. Notably, the spotlight has shifted to recombinant TB vaccines, with notable successes in preclinical and clinical trials. Exemplifying this, ID93/GLA-SE, comprising Rv2608, Rv1813, ESXV, and ESXW, has shown promise in preclinical trials^85^. In clinical trials, GamTBvac, a fusion of Ag85a and ESAT6-CFP10, has successfully navigated through phase I^88^. Similarly, M72, housing Mtb39A, and Mtb32A have demonstrated significant potency in phase II trials^86^. These achievements underscore the efficacy of recombinant vaccines, signaling a paradigm shift towards epitope-based approaches. These methods can overcome constraints such as genetic variability, antigenic shift, and antigenic drift.

Developing a novel conventional vaccine is a complicated process as it necessitates extensive laboratory testing before a series of efficacy and safety studies, which requires significant time and expense. Immunoinformatic approaches have been employed on several occasions to develop vaccines against a variety of pathogens that are currently in the late stages of their clinical development, such as *Plasmodium falciparum*^89^, *Salmonella specie*^90^, *Streptococcus pyogene*^91^, *Brucella melitensis*^92^, *Candida tropicalis*^93^, *Staphylococcus aureus*^94^, and *Bacillus anthracis*^95^. Similarly, using the immunoinformatics approach, numerous multi-epitope vaccines have been developed to target active^96–99^ and latent TB^20,100,101^. Among these, one is DNA-based^98^, and another is a mRNA-based vaccine^99^. In another study, epitopes from immunogenic exosome vesicle proteins with pathogenic properties were selected^6^. Earlier studies have identified epitopes and antigens as promising vaccine candidates against *Mtb*. For instance, Gong et al. used a peptide-based vaccine strategy to design MP3RT, which includes six immunogenic HTL peptides and has shown enhanced immune responses in humanized mice, evidenced by increased IFN-γ and CD3^+^IFN-γ^+^ T lymphocyte levels^79^. Similarly, Shiraz et al. highlighted the potential of nitroreductase family proteins in eliciting robust immune responses due to their high antigenicity and low similarity to human proteins, aiding in the fight against nitrogen-associated stresses during the dormant stage of *Mtb*^102^. However, previous studies conducted by Shiraz et al. and Sharma et al. primarily concentrated on developing multi-epitope based vaccines by selecting antigen proteins, without explicitly addressing population coverage analysis for the designed vaccines^6,102^. In contrast, our study included population coverage analysis for the vaccine, which yielded significant results.

We have designed a multi-epitope vaccine incorporating five immunogenic antigens in response to the global pandemic and the urgent need for effective TB treatments. Each antigenic protein was evaluated for its ability to stimulate the humoral response, the innate immune response, and the cell-mediated response by anticipating the BCL, HTL, and CTL epitopes, respectively. A study also revealed that IFN-*γ* promotes a protective response against TB in the lungs of mice^103^. All the identified epitopes had an IFN-γ-inducing potential, as evidenced by a positive score from the IFN epitope webserver output. According to studies, CD8^+^ T-cells may detect peptides and release cytotoxic substances such as granzyme B and perforin, which are crucial for eradicating or killing *Mtb*^104^. The chosen epitopes were tested for the same to ensure they were antigenic enough to elicit the required immune response while also being non-allergenic and non-toxic to vaccine recipients. All chosen epitopes were combined using the proper linkers, which helps adequate protein folding and maximizes flexibility. The vaccine construct utilizes adjuvants and epitopes, with an EAAAK linker used to connect them to facilitate the development of a 3D structure. In this current study, we employed the 50S ribosomal protein L7/L12 as an adjuvant to enhance the immunogenicity of the formulated vaccine. The EAAAK peptide linker adopts a rigid α-helix conformation, offering advantages in molecular design by effectively separating functional domains^105,106^. Linkers like AAY, GPGPG, and KK connect various epitopes with specific benefits, such as GPGPG inducing HTL responses and mitigating junctional immunogenicity. The GPGPG linker is an effective technique for disrupting junctional immunogenicity, which restores the immunogenicity of individual epitopes. Livingston et al. 2002 experimentally proved this using mouse models^107^. AAY (Ala-Ala-Tyr) linker, susceptible to proteasomal cleavage, enhances multi-epitope vaccine immunogenicity by reducing junctional immunogenicity^108^. B-cell epitopes linked by the KK linker are targeted by Cathepsin B, a lysosomal protease crucial for antigenic peptide digestion and MHC-II-restricted antigen presentation. This prevents antibody formation against the linearly linked peptide sequence, reducing junctional immunogenicity and enhancing overall immunogenicity^109^. In order to further improve the construct’s ability to stimulate a robust immune response, the adjuvant called 50S ribosomal subunit L7/L12 protein was attached to the N-terminus of the construct and epitopes using the EAAAK-linker. The computational analysis further confirmed the vaccine’s non-allergenicity, non-toxicity, antigenicity, and solubility. A population coverage analysis was conducted worldwide, where 97.93% of the global population was discovered. The developed vaccine’s conformational stability was established through 3-D structural modeling and validation using the Ramachandran plot, ERRAT, and ProSA analysis.

TLR2 is crucial for defense against mycobacterial infection, triggering pro-inflammatory responses upon recognizing *Mtb* components and orchestrating effective immune defense^110,111^. TLR3’s role in *Mtb* infection involves recognizing *mycoba*cterial RNA, inducing IL-10 production, suppressing IL-12p40 synthesis, and diminishing IFNγ-producing CD4^+^ Th1 cells, contributing to disease exacerbation^112^. The protein–protein docking study revealed that the vaccine had a high affinity for the critical immune cell receptors TLR2 and TLR3 by forming multiple H-bonds and non-polar contacts. Furthermore, MD simulation studies substantially support the proposed vaccines’ ability to bind consistently to immune receptors. Additionally, the vaccine and receptors negative binding energies support the stability of the complexes in the MMPBSA study. Our immune simulation experiment showed that memory B-cells and T-cells developed, with a notable activation of helper T cells. An additional noteworthy finding was that IFN-γ and IL-2 levels increased following the initial injection and persisted at their maximum levels after multiple antigen exposures. This pattern suggests a substantial presence of Th cells, contributing to effective Ig production and a robust humoral response. In addition, previous research, as Nayak et al.^113^ and Kumari R et al.^114^, suggested a correlation between elevated B-cell counts and antibody expression (IgG1–IgG2, IgM, and IgG–IgM). However, in contrast to prior observations, the current investigation did not reveal elevated IgG1–IgG2 levels. We also observed that the designed vaccine stimulates innate immune cell growth, especially dendritic cells (DCs) and natural killer (NK) cells. The host’s defense against *Mtb* relies on DCs, NK cells, and macrophages, which phagocytose and eliminate the pathogen. Macrophages and DCs serve as crucial antigen-presenting cells, presenting mycobacterial peptides, and recognition by CD4^+^ and CD8^+^ T cells occurs through MHC II and MHC I molecules, respectively^76^. Thus, the key to developing a new generation of tuberculosis vaccine is the identification of vaccine-candidate antigens and predicting and screening these immunodominant peptides. Finally, the results of in-silico cloning indicate that the vaccine can be expressed in the microbial expression system, thus making it a potential vaccine candidate against Tuberculosis. In the realm of vaccine design, in silico design is an essential screening tool for assessing candidate vaccine synthesis and efficacy. Our computational predictions demonstrate the feasibility of synthesizing the candidate vaccine in the *E. coli* system, with alternative systems such as insect and mammalian cells considered potential challenges. Successful validation through wet laboratory experiments, proven effective in prior vaccine candidates designed in silico, underscores the significance of this approach in vaccine development. Our computational results indicate that the proposed multiepitope vaccine has the potential to produce a positive immunogenic effect in the host and to provide protective immunity against the pathogen. While we have used all possible criteria to construct a potential and safe vaccine, further experimental studies could enhance our findings.

## Conclusion

To improve TB care, more attention should be given to developing innovative vaccine targets capable of controlling TB at all phases of infection. Research on novel pharmacological targets to shorten the drug regimen is also required to address the issue of treatment resistance and effectively manage TB. Five new *Mtb* antigenic proteins have been chosen for this investigation to create a multi-epitope TB vaccine. Many immunoinformatic methods have been used to extract immunodominant and non-allergic peptides from antigenic proteins. Based on the immunological and physicochemical characteristics of the vaccine, it’s safe to assume that the vaccine will produce an adequate immune response against *Mtb*. Moreover, it is essential to investigate the expression and immunogenic response of the developed vaccine in an animal model to validate its immunogenicity.

### Supplementary Information

Supplementary Figures.

Supplementary Tables.

## Data Availability

All data generated or examined throughout this research has been included in this published article and its supplementary information files.
