# Supplementary material for: Immunoinformatics and structural aided approach to develop multi-epitope based subunit vaccine against Mycobacterium tuberculosis
Source: Sci Rep. 2024 Jul 10;14:15923. doi: 10.1038/s41598-024-66858-5 (PMC11237054; doi:10.1038/s41598-024-66858-5)
Supplement: Supplementary file 1 — Supplementary Figures. [file 41598_2024_66858_MOESM1_ESM.docx]

**Supplementary Figure:**


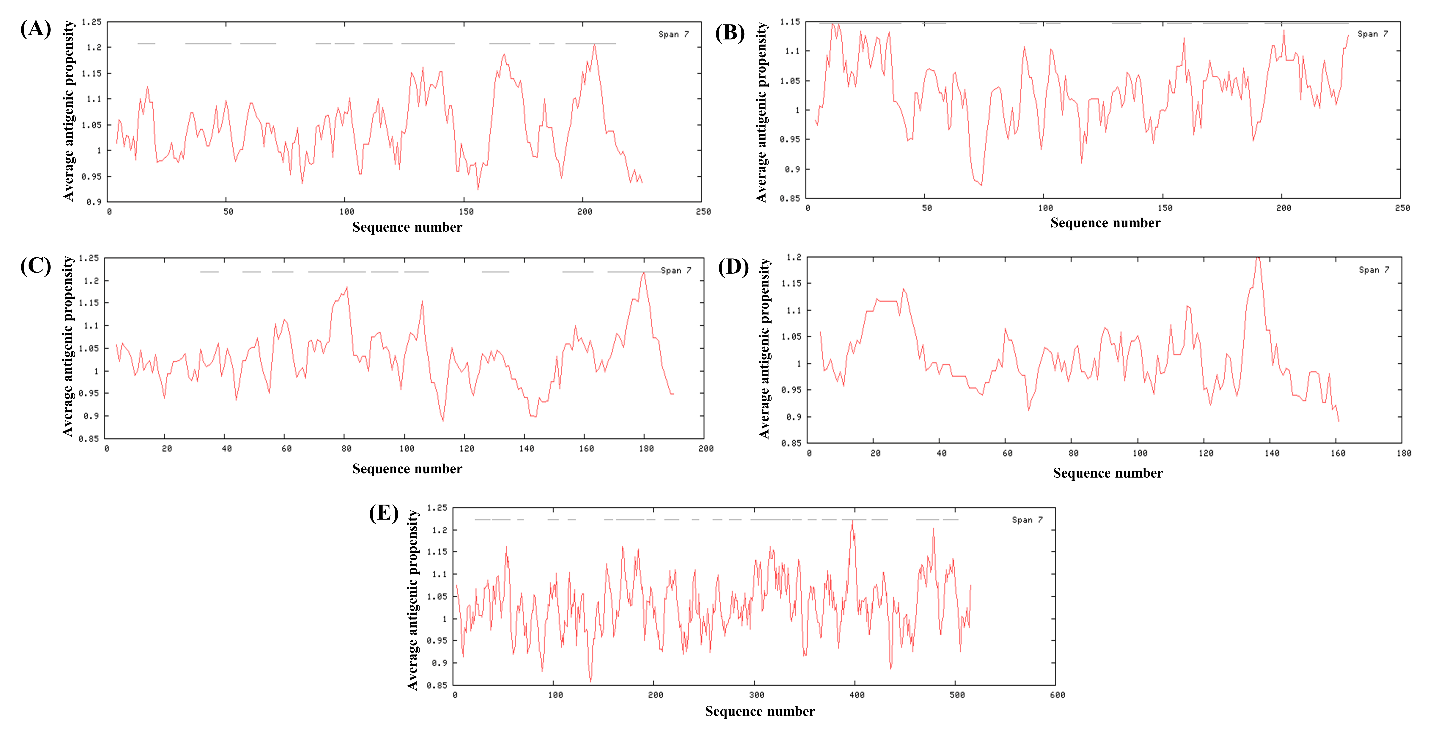


**Supplementary Figure 1.** The predicted antigenic propensity of the targeted proteins using the Antigenic Peptide Prediction tool is shown as follows: (A) Probable transcriptional regulatory protein, (B) Possible exported protein, (C) PPE family protein PPE41, (D) Conserved threonine-rich protein, and (E) Phospholipase C A. The x-axis represents the sequence number, and the y-axis represents the average antigenic propensity score. The average antigenic propensity for all selected proteins was above 1.0.


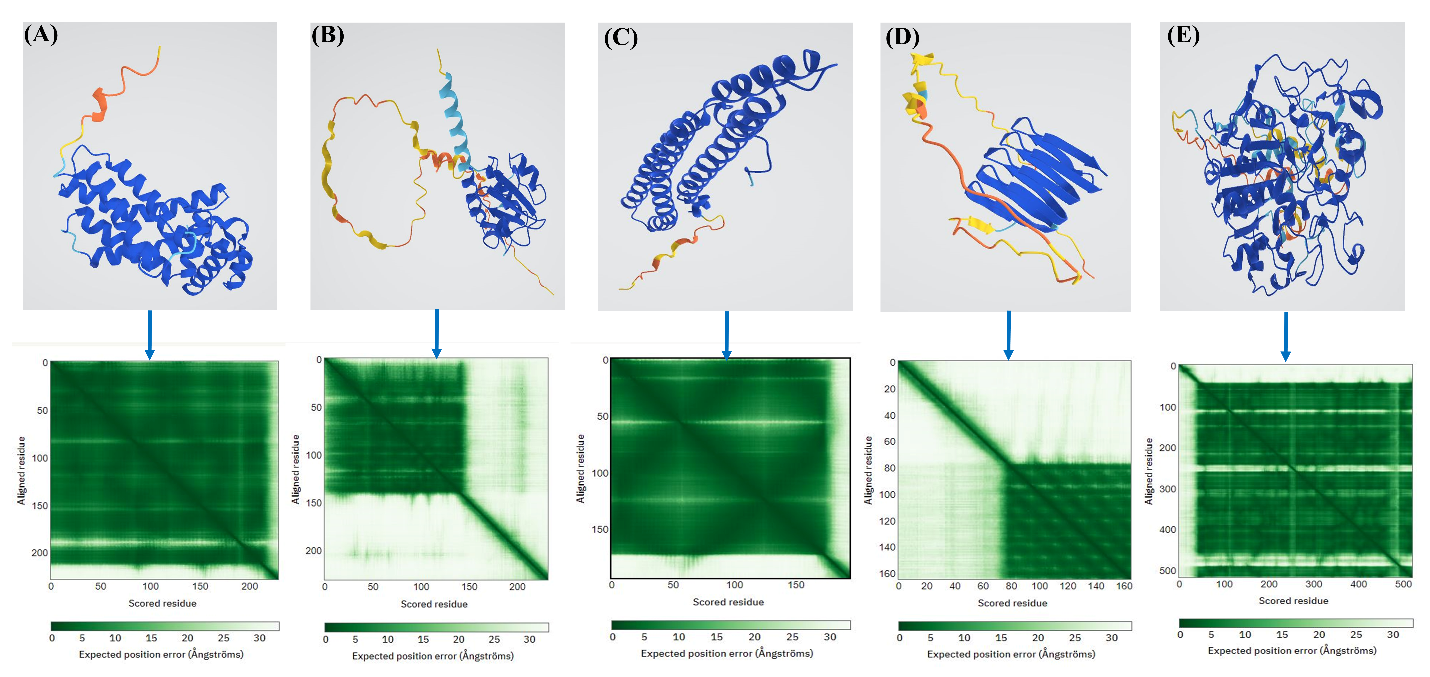


**Supplementary Figure 2.** Structural analysis of the selected five proteins by the AlphaFold program. (A) Probable transcriptional regulatory protein (B) Possible exported protein (C) PPE family protein PPE41, (D) Conserved threonine-rich protein, (E) Phospholipase C 1. The AlphaFold matrix shows the expected position error for each residue in the sequence.

**
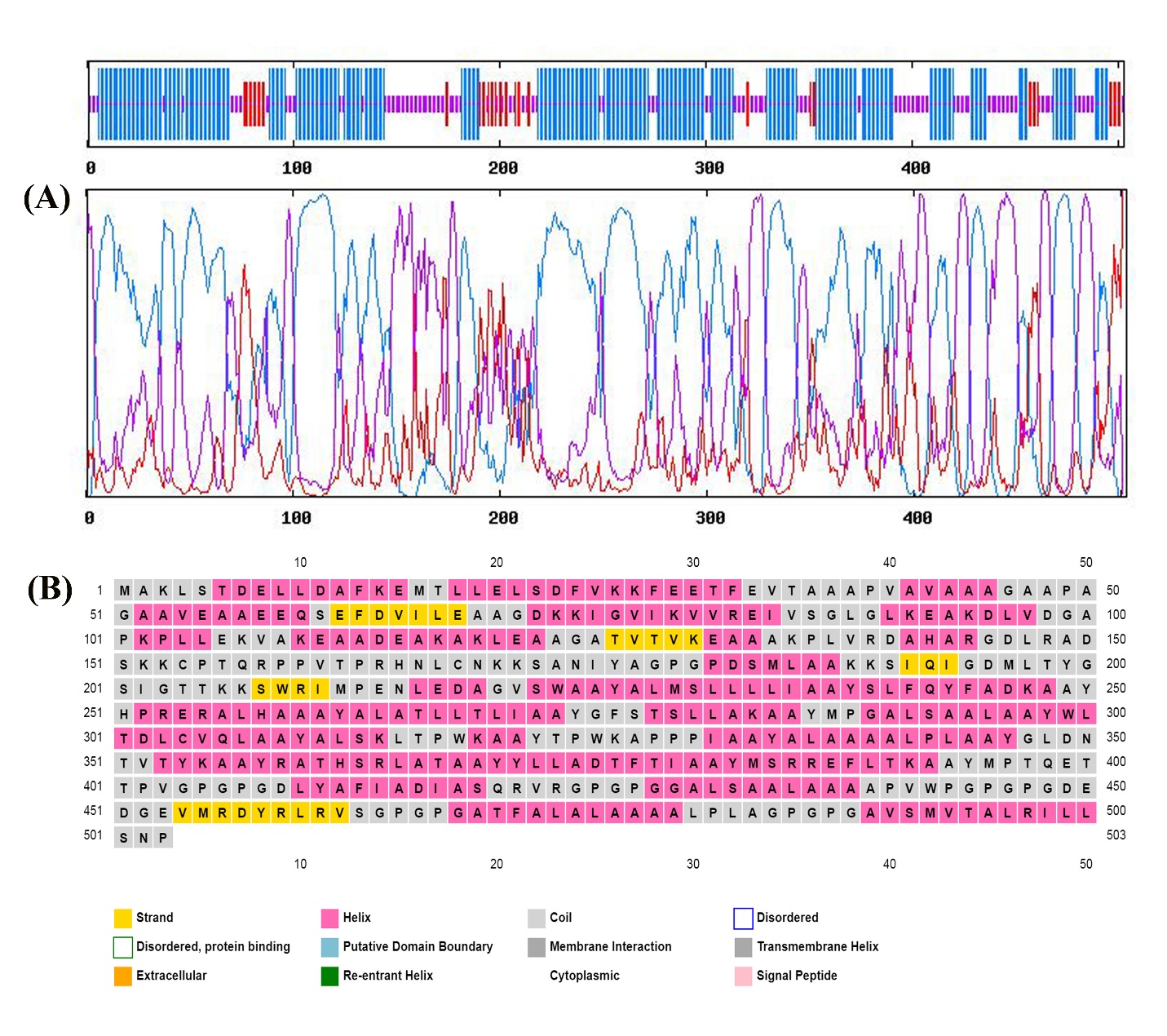
Supplementary Figure 3.** Predicted secondary structure of the multi-epitope vaccine construct: (A Secondary structure prediction from GOR IV, showing alpha helices in pink, random coils in grey, and extended strands in yellow (B) Graphical representation of the secondary structure generated by the PSIPRED server.


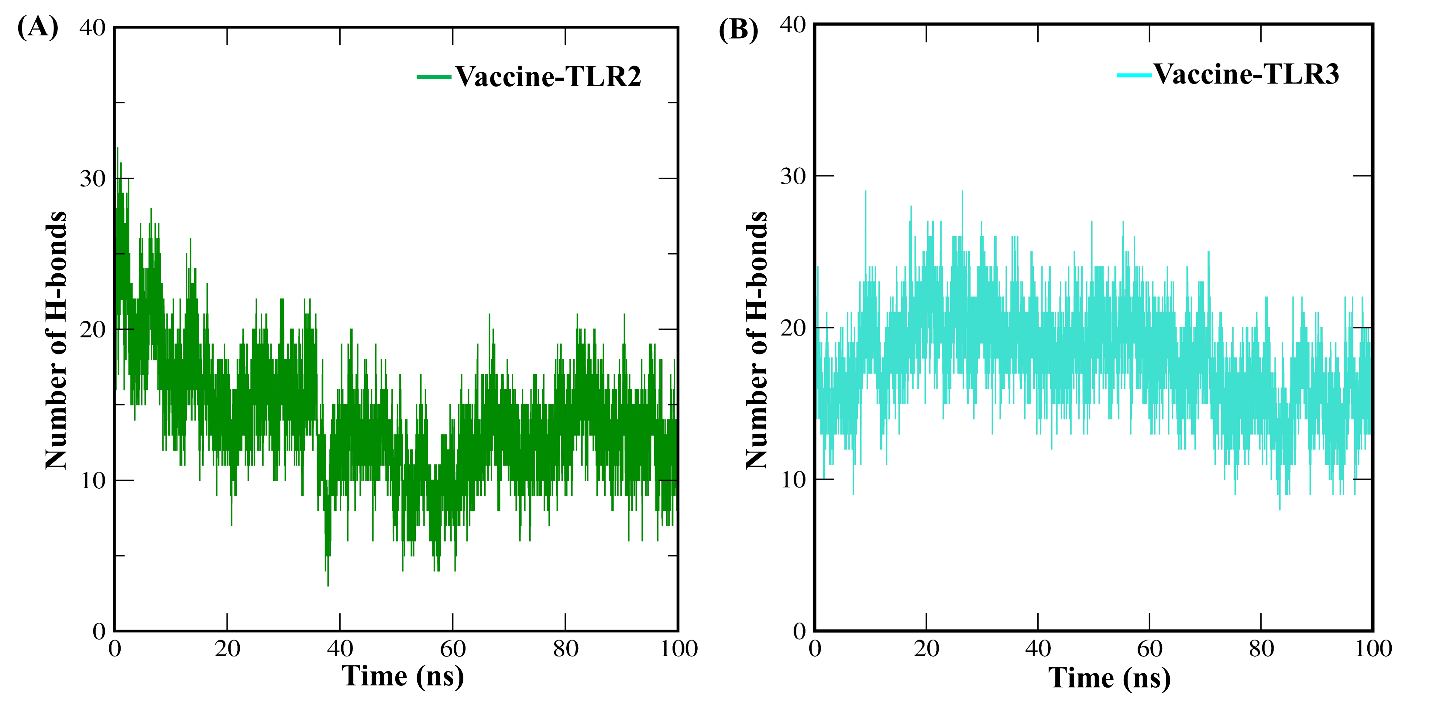


**Supplementary Figure 4.** Hydrogen bond analysis of the vaccine receptors complexes. (A)Vaccine-TLR2 complex (green) (B) Vaccine-TLR3 complex (cyan).

**
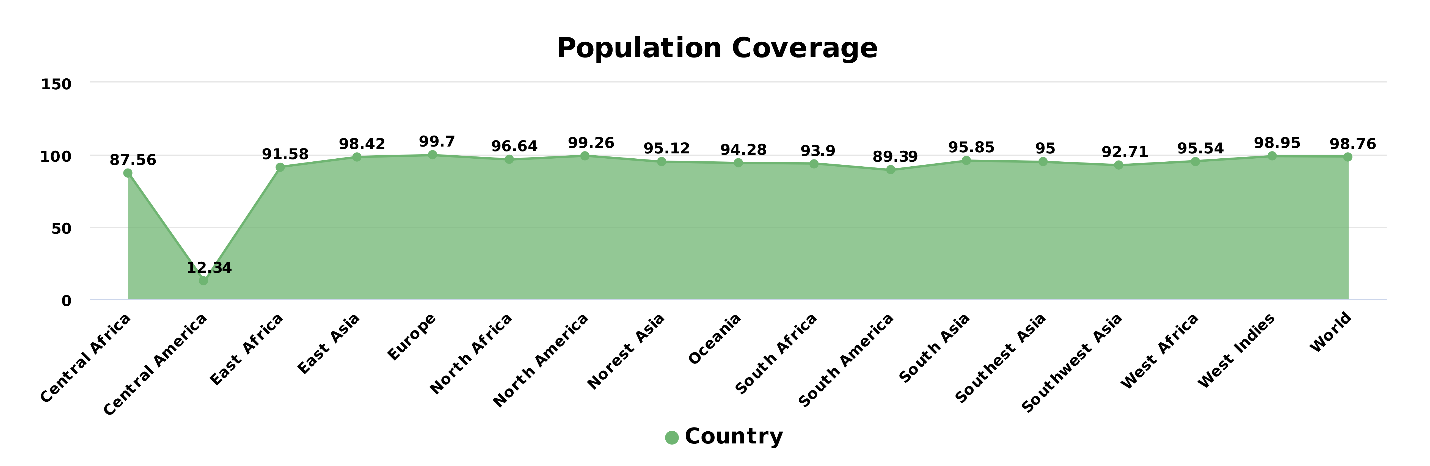
**

**Supplementary Figure 5.** The population coverage of the final multi-epitope vaccine construct was evaluated globally using the population coverage analysis tool from the IEDB database.
