# Supplementary material for: Immunoinformatics and structural aided approach to develop multi-epitope based subunit vaccine against Mycobacterium tuberculosis
Source: Sci Rep. 2024 Jul 10;14:15923. doi: 10.1038/s41598-024-66858-5 (PMC11237054; doi:10.1038/s41598-024-66858-5)
Supplement: Supplementary file 2 — Supplementary Tables. [file 41598_2024_66858_MOESM2_ESM.docx]

**Supplementary Tables:**

**Supplementary Table 1**. A list of databases, software, and web services was used to design the multi-epitope-based vaccine against *Mtb*.

| **Database, software, or web service** | **Description** | **Uniform Resource Locator (URL)** |
| --- | --- | --- |
| UniProt | Resource of protein sequence and functional information | <https://www.uniprot.org/> |
| Antigenic Peptide Prediction | Prediction of antigenicity | <http://imed.med.ucm.es/Tools/antigenic.pl> |
| AllergenFP v.1.0 | Prediction of allergenicity | <https://ddg-pharmfac.net/AllergenFP/> |
| BLASTp | Compares a protein query to a protein database | <https://blast.ncbi.nlm.nih.gov/>  [Blast.cgi?PROGRAM=blastp&PAGE_TYPE=](https://blast.ncbi.nlm.nih.gov/)  [BlastSearch&LINK_LOC=blasthome](https://blast.ncbi.nlm.nih.gov/) |
| ABCpred | Prediction of B-cell epitope | <http://crdd.osdd.net/raghava/abcpred/> |
| IEDB MHC II | Prediction of helper T-lymphocyte epitope | <https://tools.iedb.org/mhcii/> |
| NetCTL 1.2 | Prediction of cytotoxic T-lymphocyte epitope | <https://services.healthtech.dtu.dk/services/NetCTL-1.2/> |
| IFNepitope | Predicting and designing  interferon-gamma inducing epitopes | <http://crdd.osdd.net/raghava/ifnepitope/predict.php> |
| ToxinPred | Prediction of toxicity | <https://webs.iiitd.edu.in/raghava/toxinpred/> |
| ProtParam | Physicochemical properties | <https://web.expasy.org/protparam/> |
| VaxiJen v2.0 | Prediction of antigenicity | <https://www.ddg-pharmfac.net/vaxijen/VaxiJen/VaxiJen.html> |
| ANTIGENpro | Prediction of antigenicity | <https://scratch.proteomics.ics.uci.edu/> |
| SOLPro | Solubility prediction | <https://scratch.proteomics.ics.uci.edu/> |
| AllerTOP v.2.0 | Prediction of Allergenicity | <https://www.ddg-pharmfac.net/AllerTOP/> |
| AlphaFold | Tertiary structure prediction | <https://alphafold.ebi.ac.uk/> |
| PSIPRED v4.0 | Secondary structure prediction | <http://bioinf.cs.ucl.ac.uk/psipred/> |
| GOR IV | Secondary structure prediction | <https://npsa-prabi.ibcp.fr/cgi-bin/npsa_automat.pl?page=/NPSA/npsa_gor4.html> |
| RaptorX | Protein structure and function Prediction | <http://raptorx6.uchicago.edu/> |
| GalaxyRefine | Structure refinement | <https://galaxy.seoklab.org/cgi-bin/submit.cgi?type=REFINE> |
| PROCHECK | Structure validation | <https://saves.mbi.ucla.edu/> |
| ProSA-web | Structure validation | <https://prosa.services.came.sbg.ac.at/prosa.php> |
| Chimera 1.17.1 | Interactive visualization and analysis of molecular structure | <https://www.rbvi.ucsf.edu/chimera/> |
| ClusPro 2.0 | Docking study | <https://cluspro.bu.edu/login.php> |
| PDBsum | Structural interaction analysis | <https://www.ebi.ac.uk/thornton-srv/databases/pdbsum/> |
| GROMACS | A molecular dynamic package mainly designed for simulations of proteins, lipids, and nucleic acids | <https://www.gromacs.org/> |
| g_mmpbsa | A GROMACS tool for high-throughput MM-PBSA calculation | <https://rashmikumari.github.io/g_mmpbsa/> |
| IEDB population coverage | Population coverage prediction | <http://tools.iedb.org/population/> |
| JCat | Codon optimization | <https://www.jcat.de/> |
| SnapGene | *In-silico* simulation | <https://www.snapgene.com/> |
| C-ImmSim | Immune simulation | <https://kraken.iac.rm.cnr.it/C-IMMSIM/index.php> |

**Supplementary Table 2.** Physicochemical features and antigenicity, allergenicity, and solubility of the vaccine.

| **Physiochemical Properties of Vaccine** | **Values** |
| --- | --- |
| Number of amino acids | 503 |
| Molecular weight | 52.72 kDa |
| Theoretical pI | 8.15 |
| Total number of negatively charged residues (Asp + Glu) | 48 |
| Total number of positively charged residues (Arg + Lys) | 50 |
| Total number of atoms | 7455 |
| The instability index (II) is computed to be | 29.63 |
| Aliphatic Index | 94.04 |
| Grand Average of Hydropathicity (GRAVY) | 0.157 |
| Antigenicity (VaxiJen) | 0.5687 |
| Antigenicity (ANTIGENpro) | 0.5808 |
| Allergenicity (AllerTOP) | Non-allergen |
| Solubility (SOLPro) | 0.9434 |

**Supplementary Table 3.** Population coverage analysis of the constructed multi-epitope vaccine across the world.

| **Country** | **Coverage (%)** |
| --- | --- |
| Central Africa | 87.56 |
| Central America | 12.34 |
| East Africa | 91.58 |
| East Asia | 98.42 |
| Europe | 99.7 |
| North Africa | 96.64 |
| North America | 99.26 |
| Northeast Asia | 95.12 |
| Oceania | 94.28 |
| South Africa | 93.9 |
| South America | 89.39 |
| South Asia | 95.85 |
| Southeast Asia | 95 |
| Southwest Asia | 92.71 |
| West Africa | 95.54 |
| West Indies | 98.95 |
| World | 98.76 |
